# During HCV DAA Therapy Plasma Mip1B, IP10, and miRNA Profile Are Distinctly Associated with Subsequent Diagnosis of Hepatocellular Carcinoma: A Pilot Study

**DOI:** 10.3390/biology11091262

**Published:** 2022-08-25

**Authors:** Sofi Damjanovska, Hawwa Alao, Elizabeth Zebrowski, Corinne Kowal, Lenche Kostadinova, Perica Davitkov, Yngve Falck-Ytter, Carey L. Shive, Michael Cartwright, Brian Richardson, David Wald, Mark Cameron, Saba Valadkhan, Donald D. Anthony

**Affiliations:** 1 Department of Medicine, Case Western Reserve University, Cleveland, OH 44106, USA; 2 Division of Gastroenterology, VA Northeast Ohio Healthcare System, Cleveland, OH 44106, USA; 3 Rheumatology Section, VA Northeast Ohio Healthcare System, Cleveland, OH 44106, USA; 4 Department of Pathology, Case Western Reserve University, Cleveland, OH 44106, USA; 5 Population & Quantitative Health Sciences, Case Western Reserve University, Cleveland, OH 44106, USA; 6 Department of Pathology, University Hospitals Medical Center, and VA Northeast Ohio Healthcare System, Cleveland, OH 44106, USA; 7 Department of Molecular Biology and Microbiology, Case Western Reserve University, Cleveland, OH 44106, USA; 8 Division of Rheumatology, Metro Health Medical Center, Cleveland, OH 44106, USA

**Keywords:** human, hepatitis C, direct acting antiviral, hepatocellular carcinoma, micro-RNA, long non-coding RNA, immune, regulation

## Abstract

**Simple Summary:**

Hepatitis C virus (HCV) therapy lowers risk of liver cancer. MicroRNAs (miRNAs) and long non-coding RNAs (lncRNAs) regulate immune response and pathogenesis of disease. We evaluated soluble markers of interferon signaling and liver cirrhosis, plasma miRNAs and other non-coding RNAs throughout HCV therapy prior to diagnosis of liver cancer to understand factors involved in the early stages of the cancer pathogenesis. Our results of the absence of cancer pathway suppressive miRNAs, in combination with serum immune biomarkers, may help enhance ability to identify patients at high risk for liver cancer and provide timely treatments.

**Abstract:**

**Background:** Hepatitis C virus (HCV) therapy lowers risk of hepatocellular carcinoma (HCC). Little is known about factors driving/preceding HCC in treated persons. MicroRNAs (miRNAs) and long non-coding RNAs (lncRNAs) regulate host response and pathogenesis of disease. We investigated plasma levels of these RNAs and select serum markers before, during, and after HCV therapy, preceding HCC. **Methods:** Of 187 DAA treated HCV patients where therapy oriented longitudinal sampling was performed at a time without HCC diagnosis, 9 were subsequently diagnosed with HCC within 2 years of therapy. They were matched with 7 patients not diagnosed with HCC over the same time period. RNASeq was performed on plasma, and serum was assessed for biomarkers of inflammation by ELISA. **Results:** HCC diagnosis was 19 months (6–28) after therapy start in the HCC group. 73 and 63 miRs were differentially expressed at baseline (before DAA therapy) and 12 weeks after DAA therapy comparing HCC and non-HCC groups. Several lncRNA- showed differential expression as well. Several miRNA suppressors of cancer-related pathways, lncRNA- and mRNA-derived stabilized short RNAs were consistently absent in the plasma of patients who developed HCC. Serum IP10, and MCP-1 level was higher in the HCC group 12 weeks after therapy, and distinct miRNAs correlated with IP10 and MCP-1. Finally, in a focused analysis of 8 miRNAs best associated with HCC we observed expression of mi576 and mi-5189 correlation with expression of a select group of PBMC mRNA. **Conclusions:** These results are consistent with complex interplay between RNA-mediated host immune regulation and cancer suppression, strikingly skewed 12 weeks following therapy, prior to HCC diagnosis.

## 1. Introduction

Hepatocellular carcinoma (HCC) accounts for >80% of primary liver cancers, [1] {El-Serag, 2007, Hepatocellular carcinoma: epidemiology and molecular carcinogenesis} and is estimated to be the fourth most common cause of cancer-related death worldwide [2]. Chronic hepatitis B virus (HBV) and hepatitis C virus (HCV) infection are the most common underlying causes of HCC [3]. Successful treatment of HCV with DAA therapy leads to sustained virologic response (SVR) with an approximately relative risk reduction of HCC by 75% [4]. Surveillance for HCC is continued following SVR in those patients with greater risk for HCC, including those with advanced liver fibrosis (F3) and cirrhosis (F4), though better prognostic markers are needed [5]. HCC is an inflammation-related tumor with aggressive progression and rapid onset [6]. The prognosis of HCC is unfortunately poor, with a five-year survival rate of approximately 12.5% [7]. Treatment of refractory HCC is difficult, which is one of the reasons why there is a need of better understanding the complex interplay on the immunological level. Studies in the literature have shown the importance of overexpression of immune checkpoints like programmed cell death 1 (PD-1), cytotoxic T-lymphocyte antigen 4 (CTLA-4), lymphocyte activating gene 3 protein (LAG-3), and mucin domain molecule 3 (TIM-3), which leads to HCC development [8]. Inhibition of these pathways may be the future of HCC treatment [6].

MicroRNAs (miRNAs) have been investigated for their diagnostic potential in viral hepatitis, hepatic fibrosis, and HCC [7,9,10,11]. miRNAs are small non-coding RNAs 18-25 nucleotides in length that are involved in the regulation of gene expression at a post-transcriptional level. miR-15, miR-16, let-7, and miR-34 [12] are deregulated and differentially expressed in various cancers, including gastrointestinal, urological, gynecological, and lung cancer [13]. Similarly, long noncoding RNAs (lncRNA) range from ~200 nucleotides to tens of thousands of nucleotides in length and impact cellular function through a mechanism that does not depend on coding for protein [14,15]. They can arise from dedicated genomic loci or through alternative processing of transcripts from protein-coding genes [16,17] and are thought to play important regulatory roles in various biological processes via diverse mechanisms [14,18,19]. lncRNAs have also been linked to multiple cancers, including HCC [20,21,22,23].

Innate immunity, especially type I interferons (IFNs), mediate anti-tumor effects against several neoplasms [24,25]. Interferon gamma-induced protein 10 (IP10) is increased in the serum of patients with chronic HCV infection [26]. Association between the expression of IP10 mRNA and lobular necroinflammatory activity in the liver has been demonstrated [27]. Additionally, some plasma markers of cirrhosis have been examined for their relationship with HCC. Monocyte chemoattractant protein 1 (MCP-1) levels have been associated with rapid progression to liver failure in patients with HCV [28]. Studies have shown that interleukin 6 (IL-6), interleukin 1 beta (IL-1 ß), and transforming growth factor beta (TGF- ß) are involved in inflammation and development of HCC [6,29].

We evaluated soluble markers of IFN signaling and liver cirrhosis, plasma miRNAs and other non-coding RNAs as well as PBMC RNA in the longitudinal setting of HCV DAA therapy prior to diagnosis of HCC to understand factors involved in the early stages of HCC pathogenesis.

## 2. Materials and Methods

### 2.1. Study Participants and Data Extraction

A parent cohort of HCV GT1 patients at the VA Northeast Ohio Health Care System Liver Clinic (*n* = 187) was consented to longitudinal peripheral blood sampling over the course of standard of care DAA therapy (8 or 12 weeks of direct acting antivirals). Peripheral blood serum, plasma and PBMC were collected and cryopreserved at time of therapy start (referred to as week 0 or baseline), week 4, week 8 and SVR12 (12 weeks after completion of therapy). Eight of these participants that were subsequently diagnosed with HCC were matched with 8 not diagnosed with HCC, considering age, sex, race, BMI, and transient elastography (TE) score as well as stored sample availability at the time points of study. After matching, and during data analysis, one of the 8 participants in the non-HCC group was diagnosed with HCC, so the final group size was 9 for the HCC group and 7 for the non-HCC group. A separate blood sample was attempted in those that had developed HCC for comparison to pre-HCC time points in that group only.

Charts were reviewed for demographic information, ICD9/10 diagnosis of HCC (confirmed by reviewing interdisciplinary tumor board note in medical charts), platelet count (PLT), AST (Aspartate Aminotransferase), ALT (Alanine Aminotransferase), and AFP (Alpha Fetoprotein). APRI (AST to Platelet Ratio Index) and FIB-4 (Fibrosis-4) scores were calculated. HCV surveillance is performed under standard of clinical care in our clinic by 3 phase CT scan, MRI and/or ultrasound every 6 months. Charts were reviewed for 3-phase CT scan, gadolinium enhanced MRI imaging of the liver, and ultrasound. Start date of DAA therapy and DAA regimen, as well as HCV genotype and viral load before the start of the therapy, subsequent morality and cause for mortality were extracted.

### 2.2. Markers of Immune Activation and Cirrhosis

Serum was assessed for the following inflammatory markers: soluble cluster of differentiation 14 (sCD14), sCD163, interleukin-6 (IL-6), Mac-2 binding protein (Mac2BP), Monocyte Chemoattractant Protein-1 (MCP-1), interferon-inducible protein-10 (IP10), and autotaxin (ATX) by ELISA. R&D Systems, Minneapolis, MN, USA was the manufacturer for all ELISA kits, apart from the kit used for Mac2BP. For which the manufacturer was Invitrogen, Thermo Fisher Scientific, Waltham, MA, USA.

### 2.3. Statistical Analysis

Statistical analyses were performed using SPSS for Windows v. 27.0 (IBM Corp, Armonk, NY, USA). Correlations between continuous variables were evaluated by a nonparametric measure (Spearman rank sum analysis). Group comparisons were analyzed by a nonparametric test (Mann-Whitney U test). All tests of significance were two-sided and *p* values of ≤0.05 were considered significant. To evaluate the potential of a select group of miRNAs as prognostic markers, we calculated the ROC (Receiver Operating Characteristic) curve and the ROC AUC (Area Under the Curve) for a multivariate logistic regression model using normalized miRNA expression values individually and in combination. 

### 2.4. Computational Analysis of Small RNA-Seq

RNA-Seq was performed on sequencing libraries prepared from small RNAs isolated from donor plasma using Illumina TruSeq Small RNA kits, or isolated from peripheral blood mononuclear cell (PBMC), followed by sequencing using NextSeq 550 sequencer (7.5M+ single end reads/sample, 75 cycles). Pre-processing including trimming of the reads to remove adaptor-derived sequences and those that did not pass quality control was performed using Trim Galore, which combines the power of Cutadapt [30] and FastQC to ensure optimal trimming. After a second round of quality control, the reads were aligned to the hg38 human genome assembly using transcriptomic annotations including miRNA annotations from the release 33 of Gencode using STAR [31]. Quantitation of mapped reads was performed using HTSeq [32] against both the entire transcriptome and miRNA annotations, followed by TMM normalization and differential expression tests using edgeR (QL) [33]. Both the miRNA and non-miRNA transcripts showing differential expression in pairwise comparisons (defined as > 1.5-fold change with FDR < 0.05) were visually inspected using Seqmonk read view. Non-miRNA transcripts showing evidence of differential expression of stabilized short miRNA-like species were included in the analysis. Pathway analysis was performed using miRNA targets identified using a combination of TargetScan (release 7.0), TarBase (v7.0), and mSigDB (v7.0) using the GSEA tool against the KEGG database of pathways. Bioinformatics and Research computing, Whitehead Institute of Biomedical Research), TarBase (v7.0,), and mSigDB (v7.0, Diana tools, UC San Diego, Broad Institute) using the GSEA tool against the KEGG database of pathways [34,35]. 

lncRNAs showing differential expression between the HCC group and controls were identified as detailed above and evaluated for the presence of short stabilized RNA species. Those which did give rise to such short RNAs were included in the downstream analyses. In addition to short stabilized RNA species originating from lncRNA genomic loci, other stabilized short RNAs originating from protein-coding and other non-coding RNA loci were also included in the analysis. 

To calculate the area under the curve (AUC) for the Receiver Operator Characteristic (ROC) curves, a training set was generated based on the hypothesis that the detection or lack of detection of a group of identified candidate miRNA was associated with the absence and presence, respectively, of HCC development during the follow up. The training set was used to train a logistic regression model, followed by testing using the data obtained from the donors in this study [35,36]. 

## 3. Results

### 3.1. Demographics and Laboratory Features

Clinical features at the time of HCV therapy initiation of the 16 participants (9 with subsequent HCC diagnosis, and 7 without) at our single VA center are shown in Table 1. As expected for a VA cohort, all were male, many had hypertension (81%), diabetes (63%), and were active smokers (75%). From the time of DAA start, median [IQR] of chart follow up was 4 years (IQR 2, 4) for those that developed HCC and 4 (IQR 4, 4) for those that did not. Participants were treated with 8 weeks (*n* = 3) or 12 weeks (*n* = 12) of Sofosbuvir/Ledipasvir/ribavirin, or 12 weeks (*n* = 1) of Paritaprevir/ritonavir/ombitasvir/dasabuvir/ribavirin. 

### 3.2. Small Noncoding RNA Plasma Levels Differ between Those Subsequently Diagnosed with HCC and Controls

Analysis of the pattern of small RNAs (~20 nucleotides long) by high throughput sequencing in plasma pointed to strong patterns of changes during the time course of DAA therapy in each group, and several differences between the groups. While the majority of the detected small RNAs consisted of known miRNAs, a fraction corresponded to known non-miRNA small RNA species [36] and novel stabilized miRNA-like transcripts originating from lncRNAs or protein-coding RNAs [17,37,38] or currently un-annotated miRNAs. Overall, 69 miRs were the most differentially expressed at baseline comparing those that went on to develop HCC to those that did not (Table 2). At therapy week 8, 62 miR were most differentially expressed, and at SVR12 63 miRNA were most differentially expressed comparing groups. 

Focusing on within group (HCC or controls) change in miRNA over the course of therapy, 29 and 30 miRNAs were most changed in expression at week 8- and 12-weeks following therapy completion, compared to baseline in the controls, while 64 and 58 miRNAs changed the most in expression at week 8 and 12 weeks after therapy completion compared to baseline in the HCC group (Table 2). When looking at whether plasma miRNA expression differed comparing those who were diagnosed sooner vs. later after starting therapy for HCC, we compared miRNA expression in subjects who had HCC diagnosed within 1 year, 1–2 years and 2–3 years after starting HCV therapy. Despite the small number of donors in each group, several miRNAs showed a clear trend of being expressed at higher levels in the early group. For example, 13 and 4 miRNAs showed higher expression levels in the early compared to the late group at weeks 0 of therapy and SVR12, respectively, with miR-5189 showing higher expression in the early compared to the late group in all three studied time points. In comparison, zero and one miRNA (miR-103A2) showed higher expression levels in the late group compared to the early group at week 0 and SVR12, respectively.

**Figure 1:**
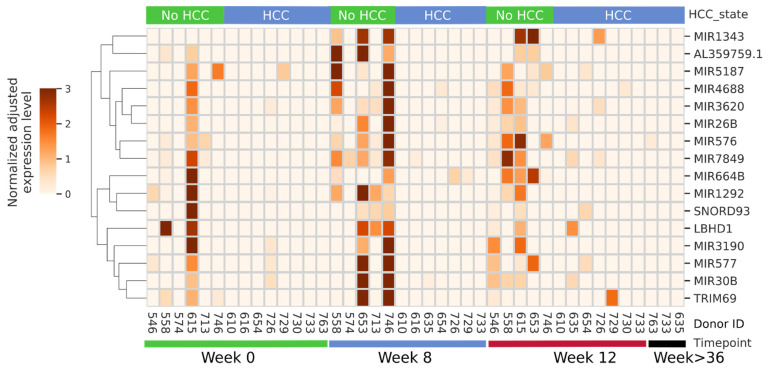
miRNAs predominantly or exclusively detected in the non-HCC group during the time course of this study. Donor IDs and the time point of each sample are indicated at the bottom, and the subsequent development of HCC, or lack of it, is shown at top. miRNA identity is shown on right, while normalized expression level key and hierarchical clustering is shown on left.

When considering specific RNAs that were the most up- or down-regulated over the course of therapy in each group we noted most differed between groups, with exception of miRNA 379 and 636 that were up-regulated in both groups (2 of 15 that were up-regulated in the controls and 2 of 14 in the HCC group) comparing SVR12 to baseline. Moreover, none of the 8 miRNAs that were down-regulated in the controls overlapped with 14 that were down-regulated in the HCC group. Given this dichotomy we next focused on miRNAs that were predominantly, or exclusively, the most expressed in each group. As shown in Figure 1, of the RNAs that are predominantly or exclusively expressed in the controls most increased at week 8 of DAA therapy, and this was in general sustained through SVR12. These small RNAs included miR-30b, miR-577, and miR-26b which have been shown to have tumor suppressor function in multiple cancers, including HCC [39,40,41,42,43]. In addition, we identified unannotated, stabilized short miRNA-like species originating from lncRNA AL359759.1, SNORD93, [36] LBHD1, and TRIM69 which were similarly most expressed in the controls at multiple study time points, while largely undetectable in the HCC group. Notably, there were no observed RNAs that were exclusively expressed in the HCC group (not shown). 

**Figure 2:**
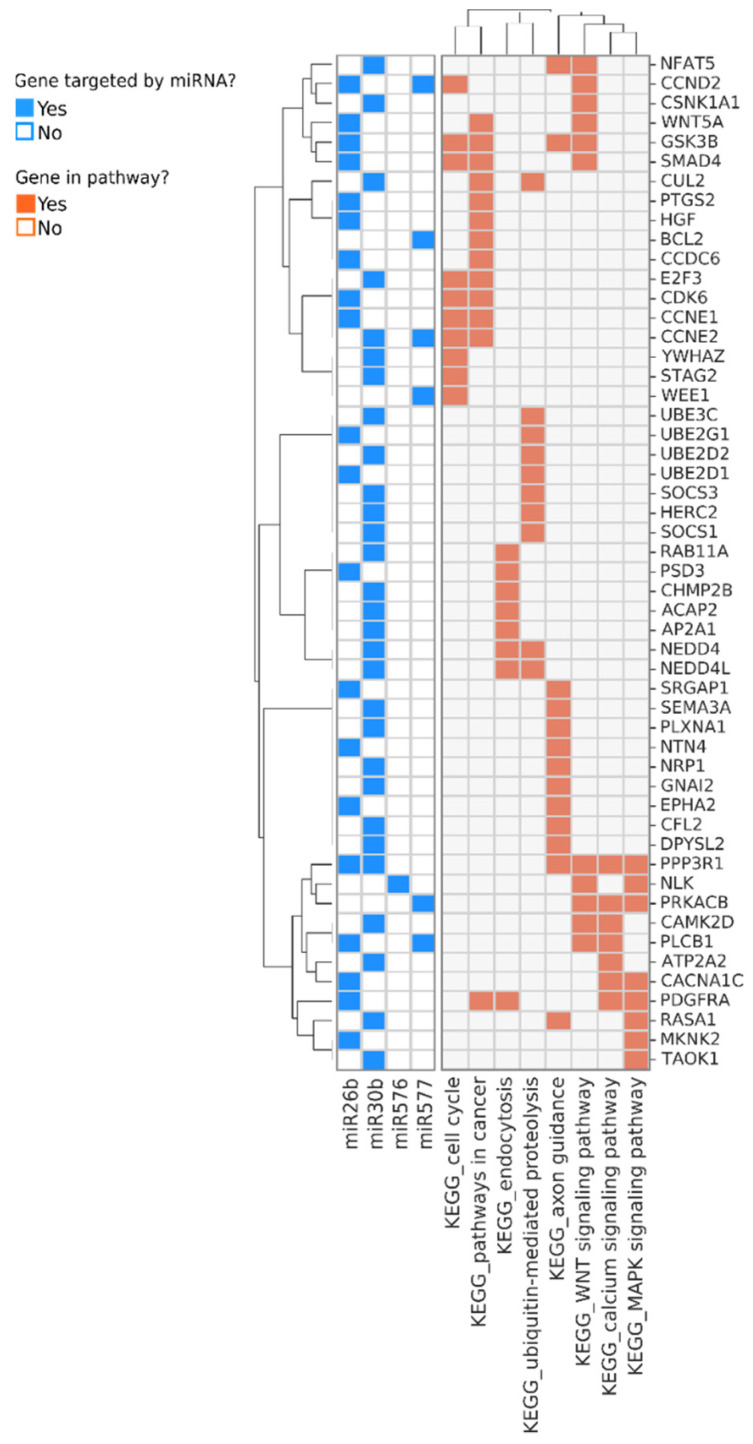
Genes and pathways regulated by the miRNAs that are predominantly or exclusively detected in samples from non-HCC donors. Genes targeted by the miRNAs are shown in rows and the most enriched pathways they participate in are shown at the bottom of the annotation matrix to the right. The annotation matrix to the left indicates the identity of the miRNAs (shown at the bottom left) which regulate each gene.

**Figure 3:**
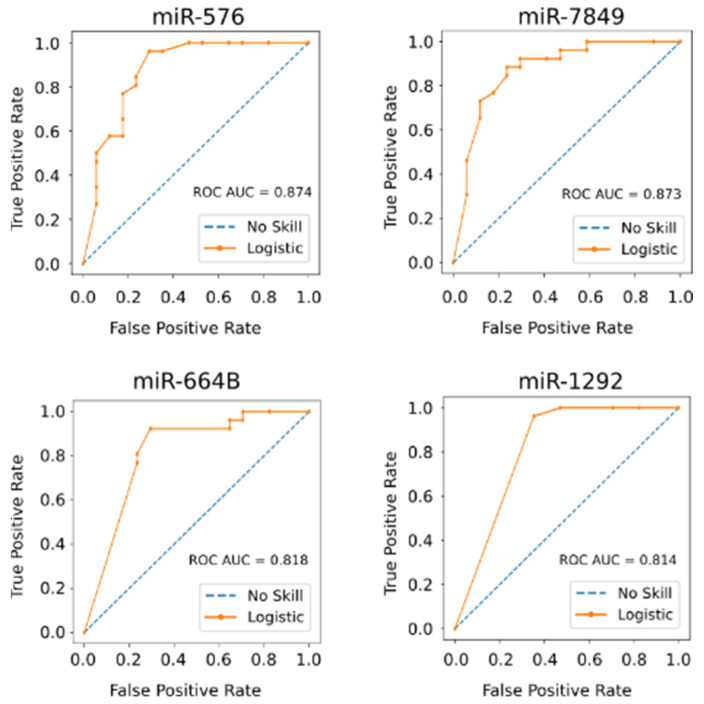
Four miRNAs show a very good ability to distinguish between the HCC and non-HCC group during treatment of HCV infection. The area under the curve (AUC) for each Receiver Operating Characteristic (ROC) curve based on logistic regression models for each RNA species was calculated. All samples throughout the time course of this study were used as test group.

Pathway analysis indicated that these small RNAs exclusively expressed in the controls suppress pathways involved in cancer, cell cycle, WNT, and MAPK signaling pathways (Figure 2). To determine the potential of these miRNAs and small stabilized RNA species to act as biomarkers for distinguishing the HCC group versus controls, we developed a logistic regression model for each RNA species, followed by calculation of receiver operating characteristic (ROC) area under the curve (AUC) (Figure 3). While nearly all miRNAs and stabilized small RNA species shown in Figure 1 had an AUC > 0.7, four miRNAs showed a very good ability (AUC > 0.8) to discriminate the HCC and control samples throughout the time points used in this study (Figure 3).

### 3.3. Biomarkers of Cirrhosis and Hepatic Inflammation

To understand the interplay between regulatory RNA and inflammatory markers observed in cirrhosis and HCC, we evaluated levels of sCD14, sCD163, IL-6, MCP-1, IP10, Mac2BP, and ATX. sCD14 is a soluble marker of monocyte/macrophage activation thought to reflect tissue inflammation. sCD163 is associated with macrophage activation and is increased in states of chronic inflammation [44]. IL-6 is an inducer of STAT3, and is a direct target of miRNA-26a and -26b, and STAT3 signaling is associated with cancer progression and metastasis [45]. MCP-1 regulates migration and infiltration of monocytes/macrophages. IP10 targets T lymphocytes, NK cells, and monocytes through CXCR3. Necroinflammatory activity in the liver associates with IP10 mRNA expression [27]. Mac2BP in glycoproteomic biomarker screening studies has been found as a noninvasive, serum glycol-marker for liver fibrosis [46,47]. ATX was initially characterized as an autocrine motility factor from A2058 melanoma cell conditioned medium. It has been subsequently shown that ATX is a marker of cirrhosis and also a potential mediator of tumorigenesis by stimulating angiogenesis, as well as survival, growth, migration, and invasion of tumor cells [48].

Within the entire patient cohort, before the start of therapy Mac2BP (r = 0.8, *p* = 0.02) and ATX positively correlated with AST level (r = 0.6, *p* = 0.03), Mac2BP and ATX positively correlated with FIB4 (r = 0.7, *p* = 0.04, and r = 0.6, *p* = 0.03), IP10 positively correlated with the viral load (r = 0.7, *p* = 0.005), and IL-6 negatively correlated with the albumin level (r = −0.6, *p* = 0.03). Several correlations were appreciated between the biomarkers of inflammation before and throughout DAA therapy as well (Table 3).

**Figure 4:**
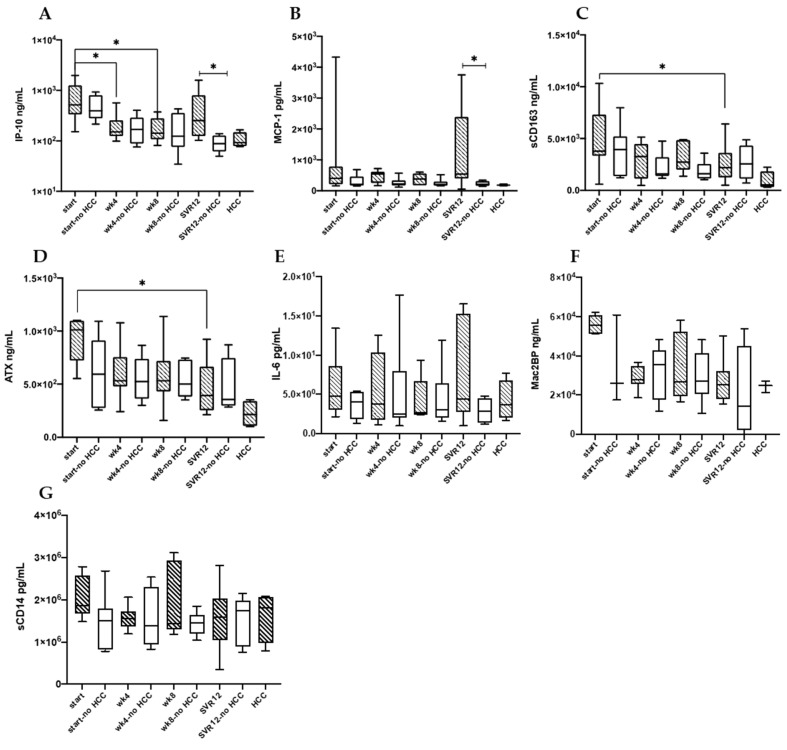
IP-10 (**A**), MCP-1 (**B**), sCD163 (**C**), ATX (**D**), IL-6 (**E**), Mac2BP (**F**), and sCD14 (**G**) serum levels are shown for HCC (*n* = 9) and no HCC (*n* = 7) groups at the beginning of DAA therapy (start and start no HCC indicating DAA therapy start for HCC and no HCC groups), through therapy (wk4, wk8), twelve weeks post-therapy (SVR12), as well as in the HCC group only once HCC developed (marked HCC). * *p* < 0.05.

When looking at sub-groups combined, sCD163 (wk0 vs. wk4 *p* = 0.006, wk0 vs. wk8 *p* = 0.02, wk0 vs. SVR12 *p* = 0.001) and ATX (wk0 vs. wk4 *p* = 0.03, wk0 vs. SVR12 *p* = 0.005) decreased over the course of therapy. Focusing on study subgroups, those that developed HCC by the end of the follow-up had a decline in sCD163 (*p* = 0.03) and ATX (*p* = 0.03) that was sustained at SVR12 (Figure 4). Over the course of therapy IP10 level decreased (wk4, *p* = 0.01; wk8, *p* = 0.01), and a decreasing trend was also appreciated in the Mac2BP levels. In the controls, there was only a trend of decreasing sCD163 and ATX levels over the course of therapy. IP10 (wk4 *p* = 0.001, wk8 0.0005, SVR12 *p* = 0.002) and Mac2BP (wk4 *p* = 0.01, wk8 0.03, SVR12 *p* = 0.03) significantly decreased over the course of therapy in the aforementioned group. In both sub-groups significant changes or trends were lacking for IL-6, sCD14, and MCP-1 level over the course of therapy.

Comparing DAA treated study participants who developed HCC to those that did not, higher levels of IP10 (*p* = 0.01), and MCP-1 (*p* = 0.04) at SVR12 were observed. There were no differences in IP10, and MCP-1 levels between the groups at the remaining time points. No other soluble markers differed between groups at any of the time points.

### 3.4. Correlations between Biomarkers of Immune Activation and miRNAs

**Figure 5:**
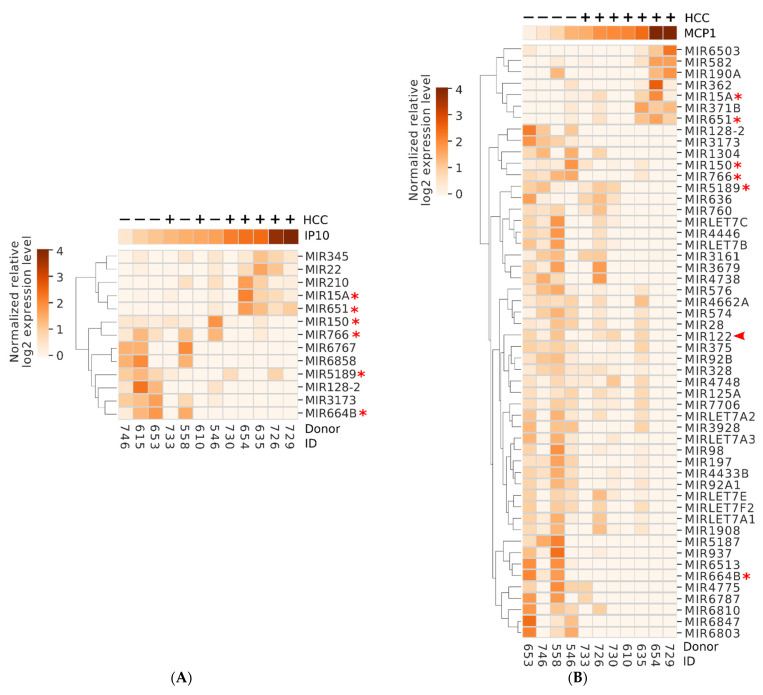
Several miRNAs show positive or negative correlation with key inflammatory markers IP10 (**A**) or MCP-1 (**B**) at 12-week SVR. Identity of each donor is shown at the bottom. The relative level of IP10/MCP-1 is shown above each heatmap using the same coloring scheme as the heatmap. Positive and negative signs at the top indicate the subsequent development or lack of HCC in each donor. Asterisks mark the miRNAs showing correlation with both MCP-1 and IP10. * indicates MIR shared between panels. Arrowhead (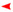) points to the liver-specific miRNA miR122.

Five miRNAs, including miR15A, miR651, and miR22, positively correlated at r value > 0.6 with serum IP10, and were more commonly observed in those that developed HCC, while eight miRNAs including miR150 and miR766, negatively correlated with IP10 levels at this time point and were more commonly observed in controls (Figure 5A). Additionally, seven miRNAs positively correlated with serum MCP-1 levels and were more commonly observed in those that developed HCC including two miRNAs (miR15A and miR651) which were also positively correlated with IP10 (Figure 5B). On the other hand, over 40 miRNAs were negatively correlated with MCP-1 levels at this time point and were more commonly observed in controls (Figure 5B), including 6 that were shared with those negatively correlated with IP10 (Figure 5B, marked by asterisks), and miR122, a liver-specific miRNA known to be downregulated in dysplastic nodules in HCV positive patients, as well as HCC due to chronic HCV infection (marked by an arrowhead) [12,49,50]. Additionally, 36 and 2 miRNAs, including the liver-specific miR-196B, were found to positively or negatively correlate with albumin level in each donor, respectively (Supplementary Figure S1). 

**Figure 6:**
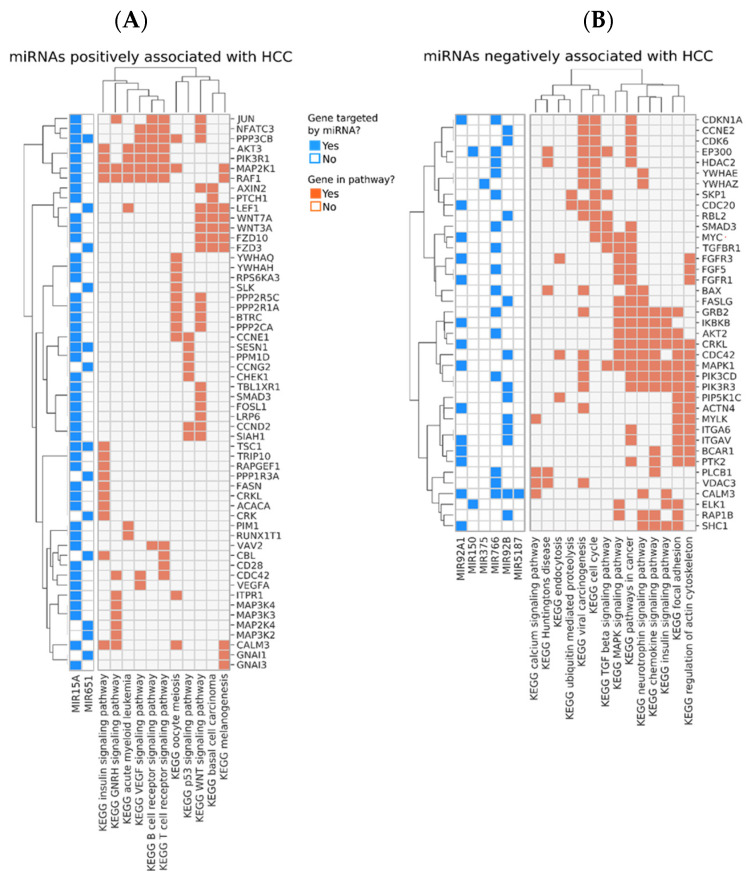
Pathways positively (**A**) or negatively (**B**) associated with the subsequent development of HCC. Genes targeted by the miRNAs are shown in rows. The annotation matrix to the left matches each miRNA (shown at the **bottom left**) with the genes they regulate (shown in rows). The annotation matrix to the right matches each gene to the most enriched pathways they participate in, which are shown at the bottom.

**Figure 7:**
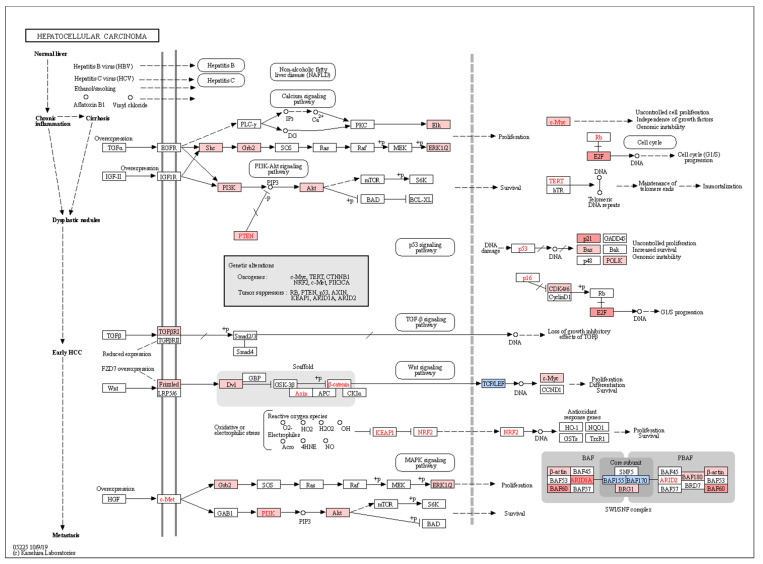
Pathways contributing to the development of hepatocellular carcinoma are differentially induced by miRNA dysregulation in HCC donors. Genes regulated by miRNAs positively or negatively enriched in HCC compared to non-HCC donors are matched to KEGG hepatocellular carcinoma pathway. Genes in blue correspond to those modulated by miRNAs associated with HCC as shown in Figure 5A, and thus, differentially downregulated in HCC donors. Conversely, genes in red are regulated by miRNAs negatively associated with HCC (Figure 5B) and thus, are differentially upregulated in HCC donors due to loss of miRNA regulation in this subset of donors.

We identified pathways which were regulated by the miRNAs that showed correlation with the level of at least two of the three biomarkers IP10, MCP-1, and albumin (Figure 5). Two miRNAs, miR15A, and miR651, were positively correlated with both IP10 and/or MCP-1, and with HCC development (Figure 5 and Figure 6A). Interestingly, the two miRNAs modulated genes belonging to pathways related to immune response, including B- and T-cell receptor signaling, and the key tumor suppressor p53, which could potentially contribute to the mechanism of oncogenesis in this group of donors (Figure 6A). On the other hand, several miRNAs were negatively correlated with IP10 and/or MCP-1, positively correlated with albumin and negatively associated with HCC. These miRNAs, in aggregate, modulated key cancer-promoting genes and pathways, including the MYC oncogene (mark by asterisk) and pathways involved in viral carcinogenesis, MAPK signaling, and cell cycle (Figure 6B and Figure 7).

### 3.5. Plasma miRNA Expression Associates with PBMC mRNA Expression

While plasma miRNA levels may derive from a different anatomic site than PBMCs (e.g., liver vs. PBMC), they may also originate from the PBMC immunocyte compartment. In the case of the latter scenario, expression of PBMC mRNAs may show correlation with plasma miRNA expression if in fact there is either co-regulation or if PBMC-derived miRNAs regulate the expression of mRNAs in these cells. We next considered the latter scenario by comparing PBMC mRNA expression profiles to plasma miRNA expression. At the SVR12 time point levels of 17 PBMC mRNA differed between HCC and non-HCC groups at adjusted false discovery rate *p* < 0.1. To understand whether PBMC mRNA expression associated with plasma miRNA levels we focused on 10 distinct miRNAs that best differentiated HCC from non-HCC groups (miR 576, 5189, 7849, 1292, 15A, 651, 150, 766, 664b and 122, Figure 3, Figure 5 and Figure 6 and Supplemental Figure S1). We observed a number of mRNAs in PBMC are associated with plasma expression of miR-576 and miR-5189 (strongest after false discovery adjustment, Table 4). Specifically, miR-576 correlated with 172 PBMC mRNA levels after false discovery adjustment, while miR-5189 correlated with 85 PBMC mRNA levels after false discovery adjustment. Notably, some mRNA expression levels are correlated with both miR-576 and miR-5189 (Supplemental Figure S2, marked with asterisk). These include NCOA4, AQP9, MPP1, GMPR, LTAF, GLUL, CTSL, CCL2, MIR22HG, TRIM58, CD83, GABARAPL1, HMOX1, NAMPT, HGB2, TREM1, PLAUR, IL8, OSM, and CXCL16. The directionality of the correlation is one where higher miRNA level is associated with lower mRNA expression and less HCC. Conversely, higher mRNA is associated with HCC, and this is associated with lower miRNA levels. At a pathway level it appears a number of chemokines or ligands or receptors are negatively and positively associated with miRNA expression and HCC, respectively. 

## 4. Discussion

We observed miRNAs and lncRNAs most differentially expressed at baseline and 12 weeks after therapy completion in persons with chronic HCV infection undergoing DAA therapy, comparing those that subsequently were diagnosed with HCC and those that were not. Several miRNA suppressors of cancer-related pathways along with lncRNA- and mRNA-derived stabilized short RNAs were consistently absent in the plasma of patients who developed HCC. Additionally, serum IP10, and MCP-1 levels were higher in the HCC group 12 weeks after therapy completion, and levels of distinct sets of miRNAs correlated with levels of IP10, MCP-1, and select peripheral blood mononuclear cell (PBMC) mRNA levels at these time points. Together, this data indicates a complex interplay between expression of non-coding RNAs, DAA therapy, serum biomarkers of liver disease and inflammation, and subsequent diagnosis of HCC.

HCV infection in hepatic cells triggers an extensive dysregulation of host miRNAs involved in regulation of the cell cycle. Of the miRs that were upregulated in the participants that developed HCC, miR21, miR15A, miR889, miR125B2, miR363, miR33B, and miR34A, have been previously described in the setting of HCV and chronic HCV complications [12,51,52,53,54,55,56]. miR-regulated pathways upregulated in the HCC group included immunologic and oncogenic signatures, statin pathway, nuclear receptors in lipid metabolism and toxicity, and SREBF and miR33 in cholesterol and lipid homeostasis. The latter pathways controlled by miR33A and miR33B.

Several lncRNAs, such as ATB (lncRNA activated by transforming factor-β) [19], DANCR (differentiation-antagonizing non-protein-coding RNA) [20], HEIH (lncRNA highly expressed in HCC) [21], MVIH (lncRNA associated with microvascular invasion in HCC), and TP73-AS1 (P73 antisense RNA 1T) [22], have been found to be dysregulated in and associated with HCC. While the use of size selected RNAs in this study prohibited us from reliably identifying differentially expressed serum lncRNAs between the two groups, short stabilized RNAs derived from lncRNAs were differentially detected in the HCC group and controls, including a lncRNA that was selectively present in the controls at all time points studied (Figure 1). Regulation of lncRNA expression may provide promising potential for cancer prevention and treatment [23,24].

Serum miRNA levels have been described as dysregulated in states of chronic liver disease and acute on chronic liver failure. Interestingly here several miRNAs correlated with IP10, MCP-1, and/or albumin levels, and those miRNAs best associated with HCC status appeared to correlate with expression of an overlapping set of PBMC mRNAs, perhaps suggesting causal relationships for expression. The cellular pathways and processes regulated by this group of miRNAs included immune-related pathways such as B- and T- cell receptor signaling, cancer suppression (p53, WNT, and VEGF signaling), HCV-mediated carcinogenesis (TGFb, MAPK, and calcium signaling), along with cell cycle regulation. As the negative regulatory impact of this group of miRNAs is absent in the HCC group, it is plausible that dysregulation of the above pathways can contribute to the development of HCC in this subgroup of HCV-infected patients. Certainly, future studies harnessing our understanding of the role of checkpoint inhibition based therapy for HCC and investigation of miRNA regulated pathways that may influence magnitude of efficacy of checkpoint inhibition may best identify the potential for select plasma or serum RNA as biomarkers or regulators of immune pathways that impact immune based therapeutic strategies [6].

A limitation of this study was that it is retrospective in nature. Another limitation is the small sample set, that included one non-HCC subject that developed HCC during the analysis, requiring group reassignment and thereby making the sample size of HCC and non-HCC groups asymmetric. Due to limited amount of cases, this study can be considered as a pilot, which will require confirmation on larger patient groups. While albumin and platelet levels did not significantly differ between groups, there were numerical differences, indicating that in part the group who developed HCC may have had more advanced liver disease. These limitations are offset by the longitudinal sampling over the course of DAA therapy in all participants, prior to diagnosis of HCC.

## 5. Conclusions

miRNA suppressors of cancer-related pathways, along with lncRNA- and mRNA-derived stabilized short RNAs, appear to be relatively absent in the plasma of patients recently treated for HCV with DAA and who are diagnosed with HCC within 6–28 months. Additionally, serum IP10, and MCP-1 levels appear higher 12 weeks after therapy in those that go on to have a diagnosis of HCC. Levels of distinct sets of miRNAs correlate with levels of IP10, MCP-1, as well as select PBMC mRNA levels. These results are consistent with a complex interplay between RNA-mediated host immune regulation and cancer suppression 12 weeks following DAA HCV therapy, and prior to HCC diagnosis.

## Figures and Tables

**Table 1:** Patient Characteristics.

	HCC	Controls	*p* < 0.05
Number	9	7	
Age (years) Median (IQR)	67 (64–70)	68 (61–70)	
Race *African American*	67%	86%	
Race *Caucasian*	33%	14%	
Sex *Male*	100%	100%	
BMI	28 (24, 29)	26 (24, 31)	
DM *n* (%)	6 (67%)	4 (57%)	
HTN *n* (%)	8 (89%)	5 (71%)	
Smoking	current 67%; former 33%	current 86%; former 14%	
Time to HCC diagnosis (month)	19 (6–28)	NA	
Fibroscan Score (kPa)			
<12.5	3	2	
≥12.5	3	3	
Not done	3 ^1^	2 ^1^	
Viral load (log^10^)	6.3 (6, 6.8)	6.2 (5.9, 6.4)	
AFP (ng/mL)	13.6 (6.5, 28.5)	13.8 (6.7, 13.9)	
AST (U/L)	75 (67, 116)	42 (35, 98)	
ALT (U/L)	110 (44, 129)	49 (34, 99)	
Albumin (g/dL)	3.4 (3.1, 3.5)	3.8 (3.5, 3.9)	
PLT (×10^3^/mm^3^)	118 (115, 171)	179 (144, 266)	
APRI	0.62 (0.5, 0.66)	0.5 (0.39, 1.1)	
Fibrosis on Radiology Exam			
US (nothing pertinent)	3	2	
US (nodularity)	2	0	
CT and/or MRI (no nodularity)	2	2	
CT and/or MRI (nodularity)	4	3	
HAPE ^2^ on Radiology Exam			
CT and/or MRI (no HAPE)	1	5	
CT and/or MRI (HAPE)	5	0	
Follow-up (years, median IQR)	4 (2, 4)	4 (4, 4)	*p* = 0.015 Fisher’s exact

^1^ Fibroscan study was not performed because the subjects had already diagnosed cirrhosis (on CT or MRI study). ^2^ HAPE—hepatic arterial phase-enhancing.

**Table 2:** Differentially Expressed Genes in Both Groups (HCC and Controls).

	*n* of DEGs with *p* ≤ 0.05	Adjusted *p* ≤ 0.10
Week 8 vs. Start Controls	29	0
Week 12 vs. Week 8 Controls	9	0
Week 12 vs. Start Controls	30	5
Week 8 vs. Start HCC	64	15
Week 12 vs. Week 8 HCC	66	13
Week 12 vs. Start HCC	58	25
HCC vs. Controls Start	69	7
HCC vs. Controls Week 8	62	7
HCC vs. Controls Week 12	63	1

**Table 3:** Correlations between biomarkers At the Start of Therapy.

**Inflammatory Marker**	**sCD163**	**ATX**	**sCD14**	**IL6**	**Mac2BP**	**IP10**	**MCP−1**	**ALT**	**AST**	**Alb**	**FIB−4**	**viral load**	**Age**	**AFP**
**sCD163**		r = 0.6 *p* = 0.06	r = 0.3 *p* = 0.4	**r = 0.6** ***p* = 0.04**	**r = 0.8** ***p* = 0.008**	r = 0.3 *p* = 0.3	r = 0.3 *p* = 0.2	r = 0.2 *p* = 0.3	r = 0.4 *p* = 0.09	r = −0.1 *p* = 0.6	r = 0.3 *p* = 0.2	r = 0.2 *p* = 0.4	r = −0.4 *p* = 0.1	r = −0.3 *p* = 0.3
**ATX**			r = 0.4 *p* = 0.3	r = 0.2 *p* = 0.5	r = 0.5 *p* = 0.2	r= −0.2 *p* = 0.6	r = 0.2 *p* = 0.5	r = 0.5 *p* = 0.1	**r = 0.6** ***p* = 0.02**	r = −0.5 *p* = 0.1	**r = 0.6** ***p* = 0.03**	r= −0.3 *p* = 0.4	r = −0.3 *p* = 0.3	**r = 0.6** ***p* = 0.05**
**sCD14**				r = 0.3 *p* = 0.3	r = 0.3 *p* = 0.5	r = 0.5 *p* = 0.1	r = 0.4 *p* = 0.2	r = 0.4 *p* = 0.2	r = 0.4 *p* = 0.1	r = −0.5 *p* = 0.09	r = 0.2 *p* = 0.5	r = 0.3 *p* = 0.3	r = 0.1 *p* = 0.2	r = 0.2 *p* = 0.5
**IL6**					r = 0.6 *p* = 0.06	r = 0.05 *p* = 0.9	r = 0.5 *p* = 0.1	r = 0.01 *p* = 1	r = 0.3 *p* = 0.3	**r = −0.6** ***p* = 0.03**	r = 0.2 *p* = 0.4	r = −0.09 *p* = 0.8	r = −0.4 *p* = 0.1	r = 0.3 *p* = 0.3
**Mac2BP**						r = 0.4 *p* = 0.3	r = 0.6 *p* = 0.1	r = 0.1 *p* = 0.6	r = 0.2 *p* = 0.4	**r = −0.6** ***p* = 0.05**	r = 0.2 *p* = 0.6	r= −0.2 *p* = 0.5	r = −0.3 *p* = 0.2	r = −0.03 *p* = 0.9
**IP10**							r = 0.4 *p* = 0.1	r = 0.3 *p* = 0.2	r = 0.3 *p* = 0.3	r = −0.2 *p* = 0.5	r = 0.1 *p* = 0.6	**r = 0.7** ***p* = 0.004**	r = −0.2 *p* = 0.4	r = −0.2 *p* = 0.5
**MCP−1**								r = 0.1 *p* = 0.6	r = 0.2 *p* = 0.4	**r = −0.6** ***p* = 0.05**	r = 0.2 *p* = 0.6	r= −0.2 *p* = 0.5	r = −0.03 *p* = 0.2	r = −0.03 *p* = 0.9
**ALT**									**r = 0.8** ***p* < 0.001**	r = −0.2 *p* = 0.4	r = 0.4 *p* = 0.1	r = 0.4 *p* = 0.09	**r = −0.5** ***p* = 0.04**	r = 0.08 *p* = 0.7
**AST**										r = −0.5 *p* = 0.06	**r = 0.8** ***p* = 0.001**	r = 0.4 *p* = 0.1	r = −0.4 *p* = 0.1	r = 0.4 *p* = 0.1
**Alb**											**r = −0.7** ***p* = 0.005**	r = 0.3 *p* = 0.7	r = 0.06 *p* = 0.8	**r = −0.6** ***p* = 0.03**
**FIB−4**												r = 0.2 *p* = 0.4	r = −0.08 *p* = 0.7	**r = 0.5** ***p* = 0.05**
**viral load**													r = −0.3 *p* = 0.2	r = −0.04 *p* = 0.8
**Age**														r = −0.07 *p* = 0.7
**AFP**														
SVR12														
**Inflammatory Marker**	**sCD163**	**ATX**	**sCD14**	**IL6**	**Mac2BP**	**IP10**	**MCP−1**	**ALT**	**AST**	**Alb**	**FIB−4**	**viral load**	**Age**	**AFP**
**sCD163**		r = 0.2 *p* = 0.6	**r = 0.6** ***p* = 0.04**	r = 0.3 *p* = 0.3	r = 0.5 *p* = 0.1	r = 0.3 *p* = 0.3	r = −0.04 *p* = 0.9	**r = 0.8** ***p* = 0.003**	**r = 0.8** ***p* = 0.004**	r = 0.06 *p* = 0.8	r = 0.3 *p* = 0.3	**r = 0.6** ***p* = 0.04**	r = −0.5 *p* = 0.08	r = −0.2 *p* = 0.4
**ATX**			r = 0.06 *p* = 0.9	r = 0.4 *p* = 0.2	r = 0.8 *p* = 0.008	r= −0.07 *p* = 0.8	r = 0.3 *p* = 0.3	r = −0.03 *p* = 0.9	r = 0.2 *p* = 0.4	r = −0.2 *p* = 0.5	r = 0.5 *p* = 0.1	r = 0.3 *p* = 0.3	r = −0.04 *p* = 0.8	**r = 0.6** ***p* = 0.04**
**sCD14**				r = −0.006 *p* = 0.9	r = 0.3 *p* = 0.4	r = 0.4 *p* = 0.2	r = −0.1 *p* = 0.7	r = 0.5 *p* = 0.08	**r = 0.6** ***p* = 0.03**	r = −0.2 *p* = 0.4	r = 0.4 *p* = 0.1	**r = 0.6** ***p* = 0.05**	r = 0.2 *p* = 0.4	r = −0.3 *p* = 0.2
**IL6**					r = 0.6 *p* = 0.07	r = 0.4 *p* = 0.2	r = 0.4 *p* = 0.2	r = 0.2 *p* = 0.6	r = 0.2 *p* = 0.4	**r = −0.6** ***p* = 0.05**	**r = 0.6** ***p* = 0.05**	r = 0.3 *p* = 0.3	r = −0.3 *p* = 0.4	**r = 0.6** ***p* = 0.04**
**Mac2BP**						r = 0.1 *p* = 0.8	r = 0.3 *p* = 0.3	r = 0.009 *p* = 1	r = 0.4 *p* = 0.1	r = −0.5 *p* = 0.1	r = 0.6 *p* = 0.06	r = 0.4 *p* = 0.1	r = −0.0 *p* = 1	**r = 0.7** ***p* = 0.02**
**IP10**							**r = 0.8** ***p* = 0.003**	**r = 0.7** ***p* = 0.01**	**r = 0.6** ***p* = 0.04**	r = −0.5 *p* = 0.09	r = 0.4 *p* = 0.1	r = 0.5 *p* = 0.1	r = −0.09 *p* = 0.7	r = 0 *p* = 1
**MCP−1**								r = 0.09 *p* = 0.7	r = 0.1 *p* = 0.6	**r = −0.6** ***p* = 0.03**	r = 0.2 *p* = 0.4	r = 0.3 *p* = 0.4	r = 0.1 *p* = 0.6	r = 0.4 *p* = 0.2
**ALT**									**r = 0.8** ***p* < 0.0001**	r = −0.2 *p* = 0.4	r = 0.4 *p* = 0.1	r = 0.4 *p* = 0.09	**r = −0.5** ***p* = 0.04**	r = 0.08 *p* = 0.7
**AST**										r = −0.5 *p* = 0.06	**r = 0.8** ***p* = 0.001**	r = 0.4 *p* = 0.1	r = −0.4 *p* = 0.1	r = 0.4 *p* = 0.1
**Alb**											**r = −0.7** ***p* = 0.005**	r = 0.3 *p* = 0.7	r = 0.06 *p* = 0.8	**r = −0.6** ***p* = 0.03**
**FIB−4**												r = 0.2 *p* = 0.4	r = −0.08 *p* = 0.7	**r = 0.5** ***p* = 0.05**
**viral load**													r = −0.3 *p* = 0.2	r = −0.04 *p* = 0.8
**Age**														r = −0.07 *p* = 0.7
**AFP**														

**Table 4:** Numbers of PBMC mRNA where expression correlates with select miRNA plasma levels.

# of DEGs	with *p* Value ≤ 0.05	Adjusted *p* Value ≤ 0.10
MIR576	1833	**172**
MIR7849	1848	4
MIR1292	1138	0
MIR15A	1111	0
MIR651	1273	1
MIR150	1606	0
MIR766	1441	4
MIR5189	1619	**85**

## Data Availability

The original contributions presented in the study are included in the article/Supplementary Material. Further inquiries can be directed to the corresponding author.

## Supplementary Materials

The following are available online at https://www.mdpi.com/article/10.3390/biology11091262/s1, Figure S1: miRNAs showing positive or negative correlation with albumin level at SVR12. Figure S2: PBMC mRNA heat map for 2 miRNA (Mi576 and Mi5189) that are associated with HCC and also associated with the most PBMC mRNA expression levels

## References

[1] El-Serag H.B., Rudolph K.L. (2007). Hepatocellular Carcinoma: Epidemiology and Molecular Carcinogenesis. Gastroenterology.

[2] Global Burden of Disease Cancer Collaboration (2018). Global, Regional, and National Cancer Incidence, Mortality, Years of Life Lost, Years Lived with Disability, and Disability-Adjusted Life-Years for 29 Cancer Groups, 1990 to 2016: A Systematic Analysis for the Global Burden of Disease Study. JAMA Oncol..

[3] El-Serag H.B. (2012). Epidemiology of viral hepatitis and hepatocellular carcinoma. Gastroenterology.

[4] (2013). Eradication of Hepatitis C Virus Infection and the Development of Hepatocellular Carcinoma. Ann. Intern. Med..

[5] European Association for the Study of the Liver (2018). EASL Clinical Practice Guidelines: Management of hepatocellular carcinoma. J. Hepatol..

[6] Leone P., Solimando A., Fasano R., Argentiero A., Malerba E., Buonavoglia A., Lupo L., De Re V., Silvestris N., Racan-elli V. (2021). The evolving role of immune checkpoint inhibitors in hepatocellular carcinoma treatment. Vaccines.

[7] Fiorino S., Bacchi-Reggiani M.L., Visani M., Acquaviva G., Fornelli A., Masetti M., Tura A., Grizzi F., Zanello M., Mastrangelo L. (2016). MicroRNAs as possible biomarkers for diagnosis and prognosis of hepatitis b-and c-related-hepatocellularcarcinoma. World J. Gastroenterol..

[8] Eggert T., Greten T.F. (2017). Tumor regulation of the tissue environment in the liver. Pharmacol. Ther..

[9] Afonso M., Rodrigues P., Simão A., Castro R.E. (2016). Circulating microRNAs as Potential Biomarkers in Non-Alcoholic Fatty Liver Disease and Hepatocellular Carcinoma. J. Clin. Med..

[10] Chauhan R., Lahiri N. (2016). Tissue- and Serum-Associated Biomarkers of Hepatocellular Carcinoma. Biomark. Cancer.

[11] Bai X., Liu Z., Shao X., Wang D., Dong E., Wang Y., Wu C.I., Yuan Y., Lu X., Li C. (2019). The heterogeneity of plasma miRNA profiles in hepatocellular carcinoma patients and the exploration of diagnostic circulating miRNAs for hepatocellular carcinoma. PLoS ONE.

[12] Ozawa T., Kandimalla R., Gao F., Nozawa H., Hata K., Nagata H., Okada S., Izumi D., Baba H., Fleshman J. (2018). A MicroRNA Signature Associated with Metastasis of T1 Colorectal Cancers to Lymph Nodes. Gastroenterology.

[13] Osaki M., Takeshita F., Ochiya T. (2008). MicroRNAs as biomarkers and therapeutic drugs in human cancer. Biomarkers.

[14] Rinn J.L., Chang H.Y. (2020). Long Noncoding RNAs: Molecular Modalities to Organismal Functions. Annu. Rev. Biochem..

[15] Bánfai B., Jia H., Khatun J., Wood E., Risk B., Gundling W.E., Kundaje A., Gunawardena H.P., Yu Y., Xie L. (2012). Long noncoding RNAs are rarely translated in two human cell lines. Genome Res..

[16] Mattick J.S., Rinn J.L. (2015). Discovery and annotation of long noncoding RNAs. Nat. Struct. Mol. Biol..

[17] Derrien T., Johnson R., Bussotti G., Tanzer A., Djebali S., Tilgner H., Guernec G., Martin D., Merkel A., Knowles D.G. (2012). The GENCODE v7 catalog of human long noncoding RNAs: Analysis of their gene structure, evolution, and expression. Genome Res..

[18] Quinn J.J., Chang H.Y. (2016). Unique features of long non-coding RNA biogenesis and function. Nat. Rev. Genet..

[19] McDonel P., Guttman M. (2019). Approaches for understanding the mechanisms of long noncoding RNA regulation of gene expression. Cold Spring Harb. Perspect. Biol..

[20] Gibb E.A., Brown C.J., Lam W.L. (2011). The functional role of long non-coding RNA in human carcinomas. Mol. Cancer.

[21] Schmitt A.M., Chang H.Y. (2016). Long Noncoding RNAs in Cancer Pathways. Cancer Cell.

[22] Hewson C., Morris K.V., Morris K.V. (2016). Form and Function of Exosome-Associated Long Non-coding RNAs in Cancer. Long Non-Coding RNAs in Human Disease.

[23] Unfried J.P., Fortes P. (2020). Lncrnas in hcv infection and hcv-related liver disease. Int. J. Mol. Sci..

[24] Moschos S., Varanasi S., Kirkwood J.M., Platanias L.C. (2005). Interferons in the treatment of solid tumors. Cytokines and Cancer. Cancer Treatment and Research.

[25] Dunn G.P., Koebel C.M., Schreiber R.D. (2006). Interferons, immunity and cancer immunoediting. Nat. Rev. Immunol..

[26] Patzwahl R., Meier V., Ramadori G., Mihm S. (2001). Enhanced expression of interferon-regulated genes in the liver of patients with chronic hepatitis C virus infection: Detection by suppression-subtractive hybridization. J. Virol..

[27] Harvey C.E., Post J., Palladinetti P., Freeman A.J., Ffrench R.A., Kumar R., Marinos G., Lloyd A.R. (2003). Expression of the chemokine IP-10 (CXCL10) by hepatocytes in chronic hepatitis C virus infection correlates with histological severity and lobular inflammation. J. Leukoc. Biol..

[28] Farci P., Wollenberg K., Diaz G., Engle R.E., Lai M.E., Klenerman P., Purcell R.H., Pybus O.G., Alter H.J. (2012). Profibrogenic chemokines and viral evolution predict rapid progression of hepatitis C to cirrhosis. Proc. Natl. Acad. Sci. USA.

[29] Nakagawa H., Maeda S., Yoshida H., Tateishi R., Masuzaki R., Ohki T., Hayakawa Y., Kinoshita H., Yamakado M., Kato N. (2009). Serum IL-6 levels and the risk for hepatocarcinogenesis in chronic hepatitis C patients: An analysis based on gender differences. Int. J. Cancer.

[30] Saeidipour B., Bakhshi S. (2013). The relationship between organizational culture and knowledge management & their simultaneous effects on customer relation management. Adv. Environ. Biol..

[31] Dobin A., Davis C.A., Schlesinger F., Drenkow J., Zaleski C., Jha S., Batut P., Chaisson M., Gingeras T.R. (2013). STAR: Ultrafast universal RNA-seq aligner. Bioinformatics.

[32] Anders S., Pyl P.T., Huber W. (2015). HTSeq—A Python framework to work with high-throughput sequencing data. Bioinformatics.

[33] Robinson M.D., McCarthy D.J., Smyth G.K. (2010). EdgeR: A Bioconductor package for differential expression analysis of digital gene expression data. Bioinformatics.

[34] Patterson D.G., Roberts J.T., King V.M., Houserova D., Barnhill E.C., Crucello A., Polska C.J., Brantley L.W., Kaufman G.C., Nguyen M. (2017). Human snoRNA-93 is processed into a microRNA-like RNA that promotes breast cancer cell invasion. NPJ Breast Cancer.

[35] Dhir A., Dhir S., Proudfoot N.J., Jopling C.L. (2015). Microprocessor mediates transcriptional termination of long noncoding RNA transcripts hosting microRNAs. Nat. Struct. Mol. Biol..

[36] Djebali S., Davis C.A., Merkel A., Dobin A., Lassmann T., Mortazavi A., Tanzer A., Lagarde J., Lin W., Schlesinger F. (2012). Landscape of transcription in human cells. Nature.

[37] Sun Z., Li A., Yu Z., Li X., Guo X., Chen R. (2017). MicroRNA-497-5p Suppresses Tumor Cell Growth of Osteosarcoma by Targeting ADP Ribosylation Factor-Like Protein 2. Cancer Biother Radiopharm..

[38] Liu Z., Wei X., Zhang A., Li C., Bai J., Dong J. (2016). Long non-coding RNA HNF1A-AS1 functioned as an oncogene and autophagy promoter in hepatocellular carcinoma through sponging hsa-miR-30b-5p. Biochem. Biophys Res. Commun..

[39] Ren X., Wang C., Xie B., Hu L., Chai H., Ding L., Tang L., Xia Y., Dou X. (2017). Tanshinone IIA induced cell death via miR30b-p53-PTPN11/SHP2 signaling pathway in human hepatocellular carcinoma cells. Eur. J. Pharmacol..

[40] Sun Q., Yu R., Wang C., Yao J., Zhang L. (2020). Circular RNA circ-CSPP1 regulates CCNE2 to facilitate hepatocellular carcinoma cell growth via sponging miR-577. Cancer Cell Int..

[41] Song K.-W., Zhang Q.-G., Tan W.-B., Fang Y.-N. (2020). Diagnostic significance of serum miR-26b and miR-21 expressions in ovarian cancer and their associations with clinicopathological characteristics and prognosis of patients. Eur. Rev. Med. Pharmacol. Sci..

[42] Grønbæk H., Sandahl T.D., Mortensen C., Vilstrup H., Møller H.J., Møller S. (2012). Soluble CD163, a marker of Kupffer cell activation, is related to portal hypertension in patients with liver cirrhosis. Aliment. Pharmacol. Ther..

[43] Yang X., Liang L., Zhang X.-F., Jia H.L., Qin Y., Zhu X.C., Gao X.M., Qiao P., Zheng Y., Sheng Y.Y. (2013). MicroRNA-26a suppresses tumor growth and metastasis of human hepatocellular carcinoma by targeting interleukin-6-Stat3 pathway. Hepatology.

[44] Kuno A., Ikehara Y., Tanaka Y., Ito K., Matsuda A., Sekiya S., Hige S., Sakamoto M., Kage M., Mizokami M. (2013). A serum “sweet-doughnut” protein facilitates fibrosis evaluation and therapy assessment in patients with viral hepatitis. Sci. Rep..

[45] Kuno A., Sato T., Shimazaki H., Unno S., Saitou K., Kiyohara K., Sogabe M., Tsuruno C., Takahama Y., Ikehara Y. (2013). Reconstruction of a robust glycodiagnostic agent supported by multiple lectin-assisted glycan profiling. Proteom.—Clin. Appl..

[46] Nam S.W., Clair T., Kim Y.S., McMarlin A., Schiffmann E., A Liotta L., Stracke M.L. (2001). Autotaxin (NPP-2), a Metastasis-enhancing Motogen, Is an Angiogenic Factor. Cancer Res..

[47] Nakao K., Miyaaki H., Ichikawa T. (2014). Antitumor function of microRNA-122 against hepatocellular carcinoma. J. Gastroenterol..

[48] Fu X., Calin G.A. (2018). miR-122 and hepatocellular carcinoma: From molecular biology to therapeutics. EBioMedicine.

[49] Clément S., Sobolewski C., Gomes D., Rojas A., Goossens N., Conzelmann S., Calo N., Negro F., Foti M. (2019). Activation of the oncogenic miR-21-5p promotes HCV replication and steatosis induced by the viral core 3a protein. Liver Int..

[50] Dai C.Y., Tsai Y.S., Chou W.W., Liu T., Huang C.F., Wang S.C., Tsai P.C., Yeh M.L., Hsieh M.Y., Huang C.I. (2018). The IL-6/STAT3 pathway upregulates microRNA-125b expression in hepatitis C virus infection. Oncotarget.

[51] Hyrina A., Olmstead A.D., Steven P., Krajden M., Tam E., Jean F. (2017). Treatment-Induced Viral Cure of Hepatitis C Virus-Infected Patients Involves a Dynamic Interplay among three Important Molecular Players in Lipid Homeostasis: Circulating microRNA (miR)-24, miR-223, and Proprotein Convertase Subtilisin/Kexin Type 9. EBioMedicine.

[52] Li X., Zhang W., Xu K., Lu J. (2020). miR-34a promotes liver fibrosis in patients with chronic hepatitis via mediating Sirt1/p53 signaling pathway. Pathol. Res. Pract..

[53] Shehata R.H., Abdelmoneim S.S., Osman O.A., Hasanain A.F., Osama A., Abdelmoneim S.S., Toraih E.A. (2017). Deregulation of miR-34a and its chaperon Hsp70 in hepatitis C virus-induced liver cirrhosis and hepatocellular carcinoma patients. Asian Pac. J. Cancer Prev..

[54] Bharali D., Jebur H.B., Baishya D., Kumar S., Sarma M.P., Masroor M., Akhter J., A Husain S., Kar P. (2018). Expression analysis of serum microRNA-34a and microRNA-183 in hepatocellular carcinoma. Asian Pac. J. Cancer Prev..

[55] Blaya D., Pose E., Coll M., Lozano J.J., Graupera I., Schierwagen R., Jansen C., Castro P., Fernandez S., Sidorova J. (2021). Profiling circulating microRNAs in patients with cirrhosis and acute-on-chronic liver failure. JHEP Rep..

[56] Zhang J., Fan J., Zhou C., Qi Y. (2017). miR-363-5p as potential prognostic marker for hepatocellular carcinoma indicated by weighted co-expression network analysis of miRNAs and mRNA. BMC Gastroenterol..

