# Supplementary material for: During HCV DAA Therapy Plasma Mip1B, IP10, and miRNA Profile Are Distinctly Associated with Subsequent Diagnosis of Hepatocellular Carcinoma: A Pilot Study
Source: Biology (Basel). 2022 Aug 25;11(9):1262. doi: 10.3390/biology11091262 (PMC9495750; doi:10.3390/biology11091262)
Supplement: Supplementary file 1 [file biology-11-01262-s001.zip › biology-1803286-supplementary.pdf]

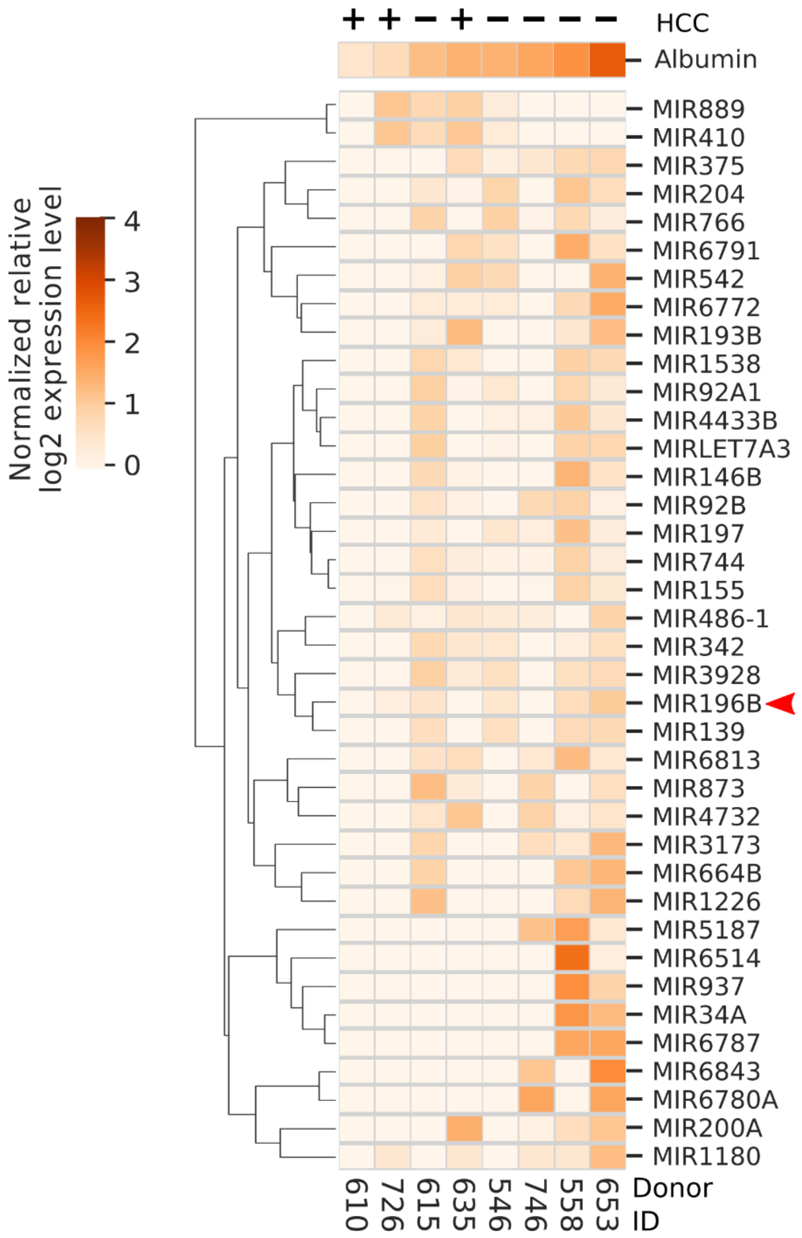

Supplementary Figure S1. miRNAs showing positive or negative correlation with albumin level at SVR12. The identity of donors is shown at the bottom. The relative level of albumin in each donor is shown at the top using the same coloring scheme as the heatmap. Positive and negative signs at the top refer to the subsequent development or lack of HCC in each donor. The liver-specific miR-196B is marked by an arrowhead.

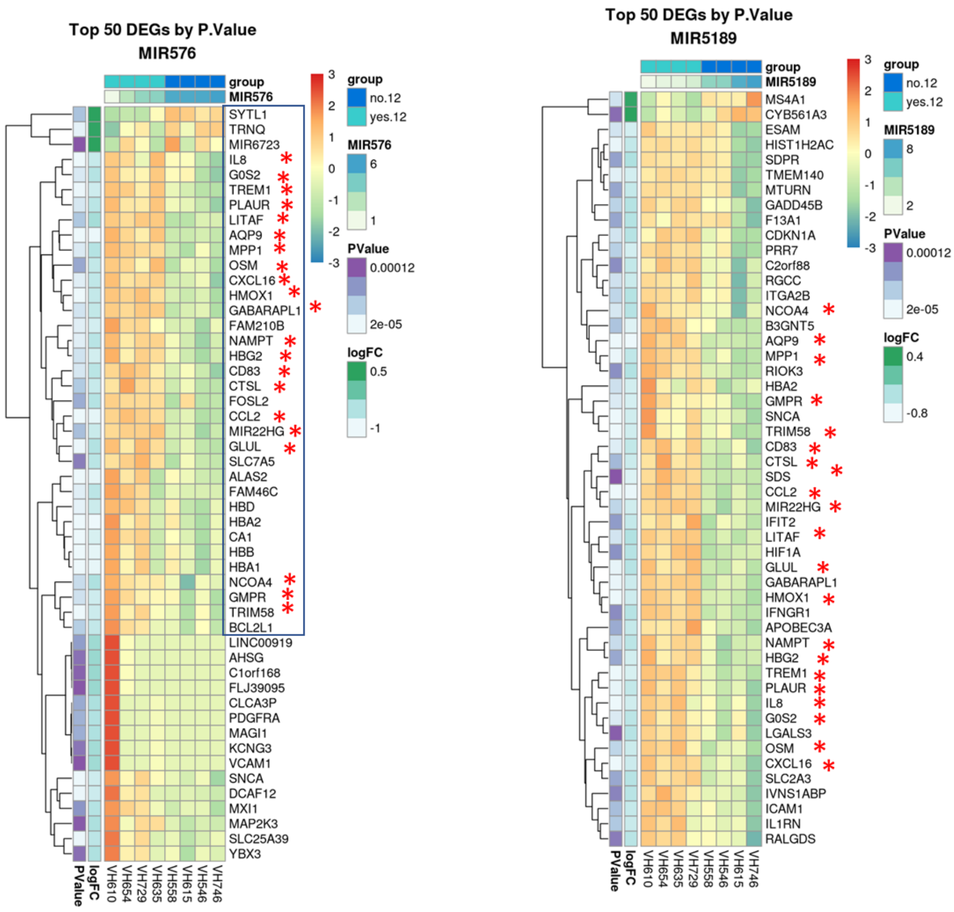

Supplementary Figure S2. For 2 miRNA (Mi576 and Mi5189) that are associated with the most PBMC mRNA expression levels (Table 4) shown here are heat map representations for those correlations in those with vs. without HCC at the SVR12 time point (when PBMC were available). Asterisks next to those mRNA that appear on both heat maps are shown (identifying NCOA4, AQP9, MPP1, GMPR, TRIM58, CD83, LTAF, GLUL, GABARAPL1, HMOX1, NAMPT, HGB2, TREM1, PLAUR, IL8, OSM, CXCL16 as common between heat maps).
